# Supplementary material for: Genetic Diversity and Phylogenetic Relationships of Castor fiber birulai in Xinjiang, China, Revealed by Mitochondrial Cytb and D-loop Sequence Analyses
Source: Animals (Basel). 2025 Jul 16;15(14):2096. doi: 10.3390/ani15142096 (PMC12291956; doi:10.3390/ani15142096)
Supplement: Supplementary file 1 [file animals-15-02096-s001.zip › animals-3718123-Figure S1 Gel electrophoresis diagram of gene amplification of Cytb and D-loop regions of partial genetic samples from Castor fiber birulai.pdf]

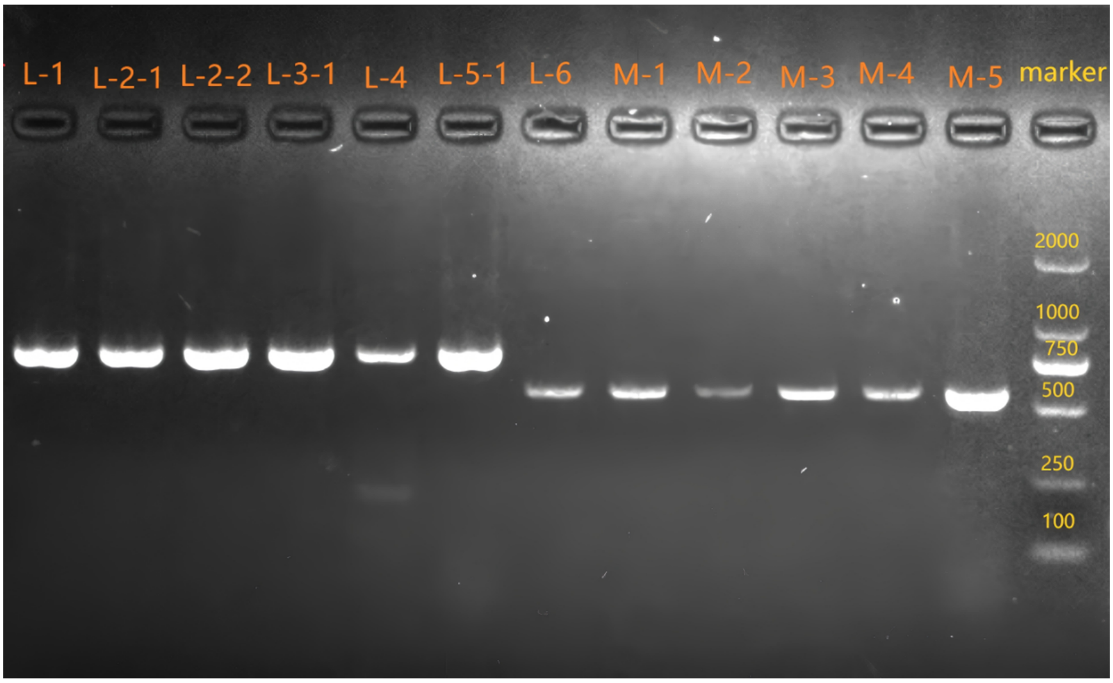

Figure S1 Gel electrophoresis diagram of gene amplification of *Cytb* and D-loop regions of partial genetic samples from *Castor fiber birulai*

Captions: Sample numbers L-1, L-2-1, L-2-2, L-3-1, L-4 and L-5-1 are the results of *Cytb* amplification(800 bp). Sample numbers L-6, M-1, M-2, M-3, M-4 and M-5 are the results of D-loop amplification(592 bp)
