# Supplementary material for: Genetic Diversity and Phylogenetic Relationships of Castor fiber birulai in Xinjiang, China, Revealed by Mitochondrial Cytb and D-loop Sequence Analyses
Source: Animals (Basel). 2025 Jul 16;15(14):2096. doi: 10.3390/ani15142096 (PMC12291956; doi:10.3390/ani15142096)
Supplement: Supplementary file 1 [file animals-15-02096-s001.zip › animals-3718123-Figure S2 Phylogenetic Tree Based on D-loop Genetic Distances among Different Castor fiber Subspecies .pdf]

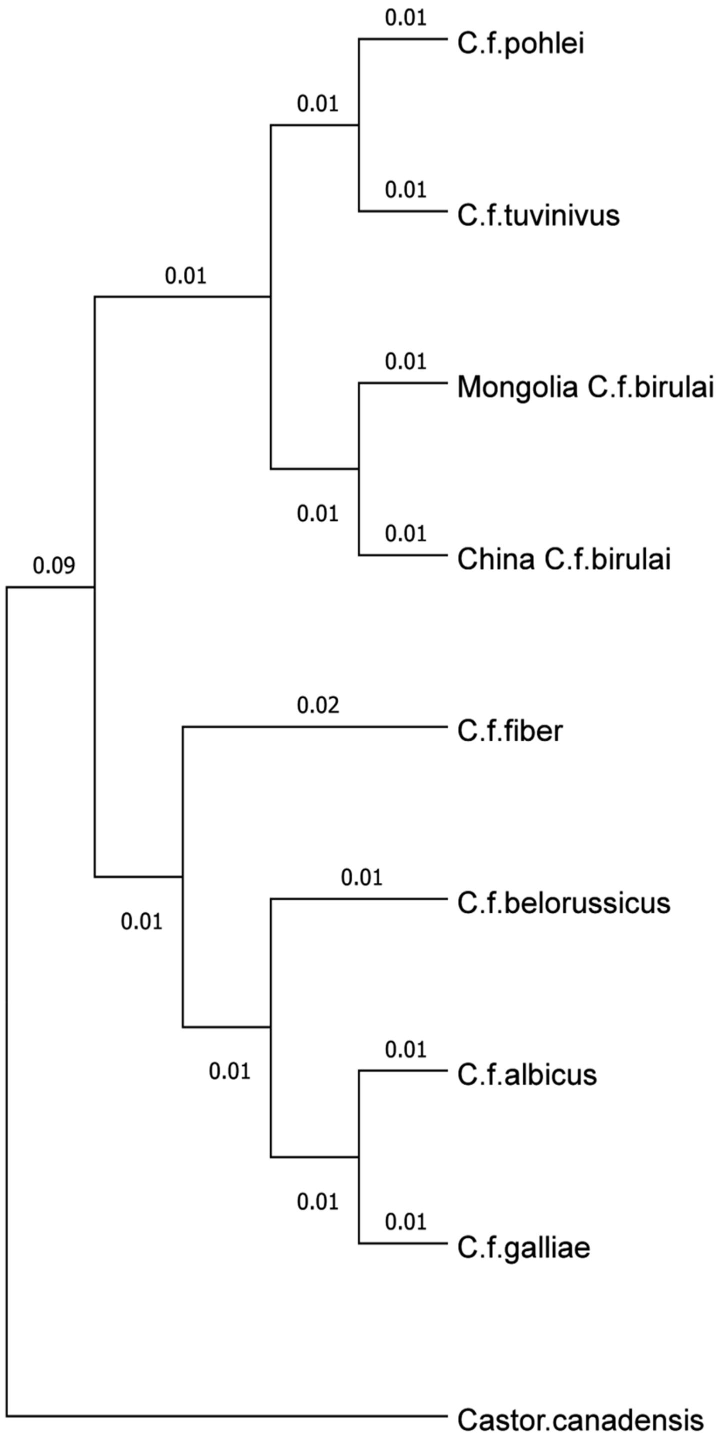

Figure S2. Phylogenetic Tree Based on D-loop Genetic Distances among Different *Castor fiber* Subspecies.
