# Supplementary material for: Genetic Diversity and Phylogenetic Relationships of Castor fiber birulai in Xinjiang, China, Revealed by Mitochondrial Cytb and D-loop Sequence Analyses
Source: Animals (Basel). 2025 Jul 16;15(14):2096. doi: 10.3390/ani15142096 (PMC12291956; doi:10.3390/ani15142096)
Supplement: Supplementary file 1 [file animals-15-02096-s001.zip › animals-3718123-Table S1. Sampling Information of the Castor fiber birulai in China.pdf]

Table S1. Sampling Information of the *Castor fiber birulai* in China

Sampling Date	Sample ID	Sample Type	Longitude	Latitude	Note
2023/3/11	L_1	Faecal sample	90.61638332	46.1831853	/
2023/3/11	L_2_1	Muscle sample	90.66684268	46.18417209	Family 1
2023/3/11	L_2_2	Faecal sample	90.66684268	46.18417209	Family 1
2023/3/11	L_3_1	Faecal sample	90.76405297	46.21381837	Family 2
2023/3/11	L_3_2	Faecal sample	90.76405297	46.21381837	Family 2
2023/3/11	L_3_3	Faecal sample	90.76405297	46.21381837	Family 2
2023/3/11	L_3_4	Faecal sample	90.76405297	46.21381837	Family 2
2023/3/11	L_3_5	Faecal sample	90.76405297	46.21381837	Family 2
2024/6/24	L_4	Faecal sample	90.92627409	46.15647333	Family 3
2025/2/4	L_5_1	Faecal sample	90.8866438	46.1669493	Family 3
2025/2/4	L_5_2	Faecal sample	90.8866438	46.1669493	Family 3
2025/2/8	L_5_3	Muscle sample	90.8866438	46.1669493	/
2025/2/20	L_6	Faecal sample	90.76405297	46.21381837	/
2024/11/5	M_1	Muscle sample	90.51976223	46.15933942	/
2024/11/5	M_2	Muscle sample	90.64744386	46.17974812	/
2024/11/5	M_3	Muscle sample	90.80281557	46.1844898	/
2024/11/5	M_4	Muscle sample	90.85883902	46.17948921	/
2024/11/5	M_5	Muscle sample	90.98644605	46.14223905	/
2024/11/5	M_6	Muscle sample	90.99864395	46.13906496	/
