# Supplementary material for: Genetic Diversity and Phylogenetic Relationships of Castor fiber birulai in Xinjiang, China, Revealed by Mitochondrial Cytb and D-loop Sequence Analyses
Source: Animals (Basel). 2025 Jul 16;15(14):2096. doi: 10.3390/ani15142096 (PMC12291956; doi:10.3390/ani15142096)
Supplement: Supplementary file 1 [file animals-15-02096-s001.zip › animals-3718123-Table S2. Sequence Information of Genetic Samples from Castor fiber and Castor canadensis Used in Phylogenetic Analys.pdf]

Table S2. Sequence Information of Genetic Samples from *Castor fiber* and *Castor canadensis* Used in Phylogenetic Analyses

number	Accession Number	Country	Species	Subspecies	Gen	Used in phylogenetic tree	*Cytb* haplo. net.	D-loop haplo. net.	Genetic distance	Source	Note（Genbank Number　）
1	DQ088706	Poland	*castor fiber*	N/A	*cytb*	*cytb*	hap_1	-	-	GenBank	
2	PV360707	Russia	*castor fiber*	N/A	*cytb*	*cytb*	hap_2	-	-	GenBank	
3	AJ389529	France	*castor fiber*	N/A	*cytb*	*cytb*	hap_2	-	-	GenBank	
4	NC_028625	Mongolia	*castor fiber*	*C.f.birulai*	*cytb*、D-loop	*cytb*、D-loop、Cytb + D-loop	hap_3	hap_14	Yes	GenBank	
5	FR691689	Russia	*castor fiber*	*C.f.pohlei*	*cytb*、D-loop	*cytb*、D-loop、Cytb + D-loop	hap_4	hap_21	Yes	GenBank	
6	FR691688	Russia	*castor fiber*	*C.f.tuvinicus*	*cytb*	*cytb*、D-loop、Cytb + D-loop	hap_5	hap_50	Yes	GenBank	
7	FR691686	Russia	*castor fiber*	*C.f.belorussicus*	*cytb*	*cytb*	hap_2	-	Yes	GenBank	
8	FR691685	Germany	*castor fiber*	*C.f.albicus*	*cytb*、D-loop	*cytb*、D-loop、Cytb + D-loop	hap_2	hap_10	Yes	GenBank	
9	DQ088708	Mongolia	*castor fiber*	*C.f.birulai*	*cytb*	*cytb*	hap_6	-	Yes	GenBank	
10	DQ088707	Russia	*castor fiber*	*C.f. pohlei*	*cytb*	*cytb*	hap_7	-	Yes	GenBank	
11	DQ088704	Germany	*castor fiber*	*C.f.albicus*	*cytb*	*cytb*	hap_2	-	Yes	GenBank	
12	DQ088705	Norway	*castor fiber*	*C.f.fiber*	*cytb*	*cytb*	hap_8	-	Yes	GenBank	
13	OQ731787	China	*castor fiber*	*C.f.birulai*	*cytb*、D-loop	*cytb*、D-loop、Cytb + D-loop	hap_3	hap_14	Yes	GenBank	
14	OQ076901	China	*castor fiber*	*C.f.birulai*	*cytb*、D-loop	*cytb*、D-loop、Cytb + D-loop	hap_3	hap_14	Yes	GenBank	
15	PQ325572	China	*castor fiber*	*C.f. birulai*	*cytb*、	*cytb*、D-loop、	hap_3	hap_14	Yes	GenBank	

					D-loop	Cytb + D-loop				
16	MG773812	United Kingdom	*castor fiber*	N/A	D-loop	D-loop	-	hap_10	-	GenBank
17	MG773811	United Kingdom	*castor fiber*	N/A	D-loop	D-loop	-	hap_15	-	GenBank
18	MG773810	United Kingdom	*castor fiber*	N/A	D-loop	D-loop	-	hap_15	-	GenBank
19	MG773809	United Kingdom	*castor fiber*	N/A	D-loop	D-loop	-	hap_10	-	GenBank
20	MG773808	United Kingdom	*castor fiber*	N/A	D-loop	D-loop	-	hap_15	-	GenBank
21	MG773807	United Kingdom	*castor fiber*	N/A	D-loop	D-loop	-	hap_10	-	GenBank
22	MG773806	United Kingdom	*castor fiber*	N/A	D-loop	D-loop	-	hap_16	-	GenBank
23	MG773805	United Kingdom	*castor fiber*	N/A	D-loop	D-loop	-	hap_10	-	GenBank
24	MG773804	United Kingdom	*castor fiber*	N/A	D-loop	D-loop	-	hap_10	-	GenBank
25	MG773803	United Kingdom	*castor fiber*	N/A	D-loop	D-loop	-	hap_17	-	GenBank
26	MG773802	United Kingdom	*castor fiber*	N/A	D-loop	D-loop	-	hap_18	-	GenBank
27	MG773801	United Kingdom	*castor fiber*	N/A	D-loop	D-loop	-	hap_13	-	GenBank
28	MG773800	United	*castor fiber*	N/A	D-loop	D-loop	-	hap_10	-	GenBank

		Kingdom								
29	MG773799	United Kingdom	*castor fiber*	N/A	D-loop	D-loop	-	hap_10	-	GenBank
30	MG773798	United Kingdom	*castor fiber*	N/A	D-loop	D-loop	-	hap_10	-	GenBank
31	MG773797	United Kingdom	*castor fiber*	N/A	D-loop	D-loop	-	hap_10	-	GenBank
32	MG773796	United Kingdom	*castor fiber*	N/A	D-loop	D-loop	-	hap_15	-	GenBank
33	KF731637	Germany	*castor fiber*	N/A	D-loop	D-loop	-	hap_19	-	GenBank
34	KF731636	Germany	*castor fiber*	N/A	D-loop	D-loop	-	hap_20	-	GenBank
35	KF731635	Germany	*castor fiber*	N/A	D-loop	D-loop	-	hap_20	-	GenBank
36	JF264888	Germany	*castor fiber*	N/A	D-loop	D-loop	-	hap_10	-	GenBank
37	JF264887	Germany	*castor fiber*	N/A	D-loop	D-loop	-	hap_21	-	GenBank
38	JF264886	Germany	*castor fiber*	N/A	D-loop	D-loop	-	hap_22	-	GenBank
39	HG915951	Czech Republik	*castor fiber*	N/A	D-loop	D-loop	-	hap_23	-	GenBank
40	HG915950	Italy	*castor fiber*	N/A	D-loop	D-loop	-	hap_24	-	GenBank
41	HG915949	Austria	*castor fiber*	N/A	D-loop	D-loop	-	hap_25	-	GenBank
42	HG915948	Austria	*castor fiber*	N/A	D-loop	D-loop	-	hap_10	-	GenBank
43	HG915947	Austria	*castor fiber*	N/A	D-loop	D-loop	-	hap_26	-	GenBank
44	HG915946	Austria	*castor fiber*	N/A	D-loop	D-loop	-	hap_27	-	GenBank
45	HG915945	Austria	*castor fiber*	N/A	D-loop	D-loop	-	hap_26	-	GenBank
46	HG915944	Austria	*castor fiber*	N/A	D-loop	D-loop	-	hap_28	-	GenBank
47	HG915943	Russia	*castor fiber*	N/A	D-loop	D-loop	-	hap_29	-	GenBank
48	HG915942	Russia	*castor fiber*	N/A	D-loop	D-loop	-	hap_19	-	GenBank

49	HG915941	Russia	*castor fiber*	N/A	D-loop	D-loop	-	hap_30	-	GenBank
50	HG915940	Russia	*castor fiber*	N/A	D-loop	D-loop	-	hap_19	-	GenBank
51	HG915939	Russia	*castor fiber*	N/A	D-loop	D-loop	-	hap_31	-	GenBank
52	HG915938	Poland	*castor fiber*	N/A	D-loop	D-loop	-	hap_32	-	GenBank
53	HG915937	Poland	*castor fiber*	N/A	D-loop	D-loop	-	hap_33	-	GenBank
54	HG915936	Poland	*castor fiber*	N/A	D-loop	D-loop	-	hap_21	-	GenBank
55	HG915935	Poland	*castor fiber*	N/A	D-loop	D-loop	-	hap_21	-	GenBank
56	HG915934	Poland	*castor fiber*	N/A	D-loop	D-loop	-	hap_21	-	GenBank
57	HG915933	Poland	*castor fiber*	N/A	D-loop	D-loop	-	hap_34	-	GenBank
58	HG915932	Poland	*castor fiber*	N/A	D-loop	D-loop	-	hap_33	-	GenBank
59	HG915931	Poland	*castor fiber*	N/A	D-loop	D-loop	-	hap_35	-	GenBank
60	HG915930	Poland	*castor fiber*	N/A	D-loop	D-loop	-	hap_35	-	GenBank
61	HG915929	Poland	*castor fiber*	N/A	D-loop	D-loop	-	hap_10	-	GenBank
62	HG915928	Germany	*castor fiber*	N/A	D-loop	D-loop	-	hap_36	-	GenBank
63	HG915927	Germany	*castor fiber*	N/A	D-loop	D-loop	-	hap_36	-	GenBank
64	HG915926	Germany	*castor fiber*	N/A	D-loop	D-loop	-	hap_23	-	GenBank
65	HG915925	Romania	*castor fiber*	N/A	D-loop	D-loop	-	hap_37	-	GenBank
66	HG915924	Romania	*castor fiber*	N/A	D-loop	D-loop	-	hap_37	-	GenBank
67	HG915923	Romania	*castor fiber*	N/A	D-loop	D-loop	-	hap_38	-	GenBank
68	HG915922	Romania	*castor fiber*	N/A	D-loop	D-loop	-	hap_37	-	GenBank
69	HG915921	Romania	*castor fiber*	N/A	D-loop	D-loop	-	hap_39	-	GenBank
70	HG915920	Norway	*castor fiber*	N/A	D-loop	D-loop	-	hap_40	-	GenBank
71	HG915919	Norway	*castor fiber*	N/A	D-loop	D-loop	-	hap_41	-	GenBank
72	HG915918	Norway	*castor fiber*	N/A	D-loop	D-loop	-	hap_42	-	GenBank
73	HG915917	Norway	*castor fiber*	N/A	D-loop	D-loop	-	hap_43	-	GenBank
74	HG915916	Norway	*castor fiber*	N/A	D-loop	D-loop	-	hap_44	-	GenBank

75	HG915915	Norway	*castor fiber*	N/A	D-loop	D-loop	-	hap_45	-	GenBank	
76	HG915914	Norway	*castor fiber*	N/A	D-loop	D-loop	-	hap_46	-	GenBank	
77	HG915913	Norway	*castor fiber*	N/A	D-loop	D-loop	-	hap_45	-	GenBank	
78	HG915912	Norway	*castor fiber*	N/A	D-loop	D-loop	-	hap_47	-	GenBank	
79	HF674458	Austria	*castor fiber*	N/A	D-loop	D-loop	-	hap_48	-	GenBank	
80	HF674457	Austria	*castor fiber*	N/A	D-loop	D-loop	-	hap_12	-	GenBank	
81	HF674456	Austria	*castor fiber*	N/A	D-loop	D-loop	-	hap_19	-	GenBank	
82	HF674455	Austria	*castor fiber*	N/A	D-loop	D-loop	-	hap_21	-	GenBank	
83	FR691689	Russia	*castor fiber*	*C.f.pohlei*	D-loop	D-loop	-	hap_49	Yes	GenBank	
84	AY623632	Mongolia	*castor fiber*	*C.f.birulai*	D-loop	D-loop	-	hap_5	Yes	GenBank	
85	AY623633	Mongolia	*castor fiber*	*C.f.birulai*	D-loop	D-loop	-	hap_5	Yes	GenBank	
86	AY623634	Mongolia	*castor fiber*	*C.f.birulai*	D-loop	D-loop	-	hap_5	Yes	GenBank	
87	AY623641	Russian	*castor fiber*	*ssp.2*	D-loop	D-loop	-	hap_3	-	GenBank	
88	AY623642	Poland	*castor fiber*	*ssp.1*	D-loop	D-loop	-	hap_4	-	GenBank	
89	AY623643	Poland	*castor fiber*	*ssp.1*	D-loop	D-loop	-	hap_3	-	GenBank	
90	AY623635	Russia	*castor fiber*	*C.f.pohlei*	D-loop	D-loop	-	hap_1	Yes	GenBank	
91	AY623636	Russia	*castor fiber*	*C.f.pohlei*	D-loop	D-loop	-	hap_2	Yes	GenBank	
92	AY623637	Russia	*castor fiber*	*C.f.tuvinicus*	D-loop	D-loop	-	hap_6	Yes	GenBank	
93	AY623638	Russia	*castor fiber*	*C.f.tuvinicus*	D-loop	D-loop	-	hap_7	Yes	GenBank	
94	AY623639	Russia	*castor fiber*	*C.f. tuvinicus*	D-loop	D-loop	-	hap_8	Yes	GenBank	
95	AY623640	Russia	*castor fiber*	*C.f.tuvinicus*	D-loop	D-loop	-	hap_9	Yes	GenBank	
96	DQ088700	Germany	*castor fiber*	*C.f. albicus*	D-loop	D-loop	-	hap_10	Yes	GenBank	
97	DQ088701	Germany	*castor fiber*	*C.f.albicus*	D-loop	D-loop	-	hap_11	Yes	GenBank	
98	DQ088702	Norway	*castor fiber*	*C.f. fiber*	D-loop	D-loop	-	hap_12	Yes	GenBank	
99	DQ088703	French	*castor fiber*	*C.f.galliae*	D-loop	D-loop	-	hap_13	Yes	GenBank	
100	L_1	China	*castor fiber*	*C.f.birulai*	*cytb*、	*cytb*、D-loop、	hap_3	hap_14	Yes	This study	*cytb*:PV776086

					D-loop	Cytb + D-loop					D-Loop:PV776067
101	L_2_1	China	*castor fiber*	*C.f.birulai*	*cytb*、D-loop	*cytb*、D-loop、Cytb + D-loop	hap_3	hap_14	Yes	This study	*cytb*:PV776087<br>D-Loop:PV776068
102	L_2_2	China	*castor fiber*	*C.f.birulai*	*cytb*、D-loop	*cytb*、D-loop、Cytb + D-loop	hap_3	hap_14	Yes	This study	*cytb*:PV776088<br>D-Loop:PV776069
103	L_3_1	China	*castor fiber*	*C.f.birulai*	*cytb*、D-loop	*cytb*、D-loop、Cytb + D-loop	hap_3	hap_14	Yes	This study	*cytb*:PV776089<br>D-Loop:PV776070
104	L_3_2	China	*castor fiber*	*C.f.birulai*	*cytb*、D-loop	*cytb*、D-loop、Cytb + D-loop	hap_3	hap_14	Yes	This study	*cytb*:PV776090<br>D-Loop:PV776071
105	L_3_3	China	*castor fiber*	*C.f.birulai*	*cytb*、D-loop	*cytb*、D-loop、Cytb + D-loop	hap_3	hap_14	Yes	This study	*cytb*:PV776091<br>D-Loop:PV776072
106	L_3_4	China	*castor fiber*	*C.f.birulai*	*cytb*、D-loop	*cytb*、D-loop、Cytb + D-loop	hap_3	hap_14	Yes	This study	*cytb*:PV776092<br>D-Loop:PV776073
107	L_3_5	China	*castor fiber*	*C.f.birulai*	*cytb*、D-loop	*cytb*、D-loop、Cytb + D-loop	hap_3	hap_14	Yes	This study	*cytb*:PV776093<br>D-Loop:PV776074
108	L_4	China	*castor fiber*	*C.f.birulai*	*cytb*、D-loop	*cytb*、D-loop、Cytb + D-loop	hap_3	hap_14	Yes	This study	*cytb*:PV776094<br>D-Loop:PV776075
109	L_5_1	China	*castor fiber*	*C.f.birulai*	*cytb*、D-loop	*cytb*、D-loop、Cytb + D-loop	hap_3	hap_52	Yes	This study	*cytb*:PV776095<br>D-Loop:PV776076
110	L_5_2	China	*castor fiber*	*C.f.birulai*	*cytb*、D-loop	*cytb*、D-loop、Cytb + D-loop	hap_3	hap_14	Yes	This study	*cytb*:PV776096<br>D-Loop:PV776077
111	L_5_3	China	*castor fiber*	*C.f.birulai*	*cytb*、D-loop	*cytb*、D-loop、Cytb + D-loop	hap_3	hap_52	Yes	This study	*cytb*:PV776097<br>D-Loop:PV776078
112	L_6	China	*castor fiber*	*C.f.birulai*	*cytb*、D-loop	*cytb*、D-loop、Cytb + D-loop	hap_3	hap_14	Yes	This study	*cytb*:PV776098<br>D-Loop:PV776079
113	M_1	China	*castor fiber*	*C.f.birulai*	*cytb*、	*cytb*、D-loop、	hap_3	hap_14	Yes	This study	*cytb*:PV776099

					D-loop	Cytb + D-loop					D-Loop:PV776080
114	M_2	China	*castor fiber*	*C.f.birulai*	*cytb*、D-loop	*cytb*、D-loop、Cytb + D-loop	hap_3	hap_14	Yes	This study	*cytb*:PV776100 D-Loop:PV776081
115	M_3	China	*castor fiber*	*C.f.birulai*	*cytb*、D-loop	*cytb*、D-loop、Cytb + D-loop	hap_3	hap_14	Yes	This study	*cytb*:PV776101 D-Loop:PV776082
116	M_4	China	*castor fiber*	*C.f.birulai*	*cytb*、D-loop	*cytb*、D-loop、Cytb + D-loop	hap_3	hap_14	Yes	This study	*cytb*:PV776102 D-Loop:PV776083
117	M_5	China	*castor fiber*	*C.f.birulai*	*cytb*、D-loop	*cytb*、D-loop、Cytb + D-loop	hap_3	hap_14	Yes	This study	*cytb*:PV776103 D-Loop:PV776084
118	M_6	China	*castor fiber*	*C.f.birulai*	*cytb*、D-loop	*cytb*、D-loop、Cytb + D-loop	hap_3	hap_14	Yes	This study	*cytb*:PV776104 D-Loop:PV776085
119	KY321562	Canada	*Castor canadensis*	N/A	*cytb*、D-loop	*cytb*、D-loop、Cytb + D-loop	-	-	-	GenBank	
120	NC_033912	Canada	*Castor canadensis*	N/A	*cytb*、D-loop	*cytb*、D-loop、Cytb + D-loop	-	-	-	GenBank	
121	KY321562	Canada	*Castor canadensis*	N/A	*cytb*、D-loop	*cytb*、D-loop、Cytb + D-loop	-	-	-	GenBank	
122	FR691684	Finland	*Castor canadensis*	N/A	*cytb*、D-loop	*cytb*、D-loop、Cytb + D-loop	-	-	-	GenBank	
123	EU476079	Argentina	*Castor canadensis*	N/A	D-loop	D-loop	-	-	-	GenBank	
124	AY968083	Argentina	*Castor canadensis*	N/A	D-loop	D-loop	-	-	-	GenBank	
125	AY787827	Argentina	*Castor canadensis*	N/A	D-loop	D-loop	-	-	-	GenBank	
126	AY787826	Argentina	*Castor*	N/A	D-loop	D-loop	-	-	-	GenBank	

			canadensis							
127	AY787825	Argentina	*Castor canadensis*	N/A	D-loop	D-loop	-	-	-	GenBank
128	AY787824	Argentina	*Castor canadensis*	N/A	D-loop	D-loop	-	-	-	GenBank
129	AY787823	Argentina	*Castor canadensis*	N/A	D-loop	D-loop	-	-	-	GenBank
130	AY787822	Argentina	*Castor canadensis*	N/A	D-loop	D-loop	-	-	-	GenBank
131	JN655159	Argentina	*Castor canadensis*	N/A	D-loop	D-loop	-	-	-	GenBank
132	JN655158	Argentina	*Castor canadensis*	N/A	D-loop	D-loop	-	-	-	GenBank
133	OR655427	South Korea	*Lutra lutra*	N/A	*cytb*、D-loop	Out group	-	-	-	GenBank
